# Promoter hypermethylation as a novel regulator of ANO1 expression and function in prostate cancer bone metastasis

**DOI:** 10.1038/s41598-024-62478-1

**Published:** 2024-05-21

**Authors:** Yonghwan Shin, Sungmin Kim, Woojin An

**Affiliations:** Department of Biochemistry and Molecular Medicine, Norris Comprehensive Cancer Center, University of Southern California, Los Angeles, CA 90033 USA

**Keywords:** ANO1, Epigenetics, DNA methylation, Metastasis, Prostate cancer, Cell biology, Cancer, Metastasis

## Abstract

Despite growing evidence implicating the calcium-activated chloride channel anoctamin1 (ANO1) in cancer metastasis, its direct impact on the metastatic potential of prostate cancer and the possible significance of epigenetic alteration in this process are not fully understood. Here, we show that ANO1 is minimally expressed in LNCap and DU145 prostate cancer cell lines with low metastatic potential but overexpressed in high metastatic PC3 prostate cancer cell line. The treatment of LNCap and DU145 cells with DNMT inhibitor 5-aza-2′-deoxycytidine (5-Aza-CdR) potentiates ANO1 expression, suggesting that DNA methylation is one of the mechanisms controlling ANO1 expression. Consistent with this notion, hypermethylation was detected at the CpG island of ANO1 promoter region in LNCap and DU145 cells, and 5-Aza-CdR treatment resulted in a drastic demethylation at promoter CpG methylation sites. Upon 5-Aza-CdR treatment, metastatic indexes, such as cell motility, invasion, and metastasis-related gene expression, were significantly altered in LNCap and DU145 cells. These 5-Aza-CdR-induced metastatic hallmarks were, however, almost completely ablated by stable knockdown of ANO1. These in vitro discoveries were further supported by our in vivo observation that ANO1 expression in xenograft mouse models enhances the metastatic dissemination of prostate cancer cells into tibial bone and the development of osteolytic lesions. Collectively, our results help elucidate the critical role of ANO1 expression in prostate cancer bone metastases, which is epigenetically modulated by promoter CpG methylation.

## Introduction

Epigenetic modifications, such as DNA methylation and histone modifications, play a crucial role in regulating genome architecture and a series of cellular reactions. These epigenetic changes can influence gene expression without altering the DNA sequence, thereby affecting heritable information in human cells^1–3^. Among known epigenetic processes, DNA methylation has been most extensively studied and shown to be most frequently associated with the development of human cancer. Altered DNA methylation occurring in the gene promoter region can influence global and site-specific gene expression by modulating the degree of chromatin condensation as well as the accessibility of transcription factors to target genes. In a wide range of cancers, DNA methyltransferases (DNMTs) such as DNMT1, DNMT3a, and DNMT3b have been shown to exhibit overexpression and initiate and maintain DNA methylation at promoter regions^1,2,4,5^. In view of these effects, DNMT inhibitors such as 5-Aza-CdR have been used to restore gene expression through DNA demethylation for certain types of cancer such as acute myeloid leukemia and myelodysplastic syndromes^6–9^. Of special relevance to the present study, promoter DNA hypermethylation was also identified as a critical step in regulating the expression of genes in the invasion-metastasis cascade, and 5-Aza-CdR treatment increases invasive/metastatic potentials of cancer cells^10^. Despite these advances, however, mechanistic views of how DNA methylation participates in these epigenetic processes and how 5-Aza-CdR treatment alters metastatic phenotype of cancer cells remain lacking.

Prostate cancer constitutes a significant health burden, being the most commonly diagnosed cancer and the second leading cause of cancer-related deaths among men in the United States^11^. Prostate cancer can be diagnosed as a localized or advanced disease, with several treatment options including surgery, radiation therapy, hormonal therapy, chemotherapy, immunotherapy, and combinations of these therapies^12,13^. Although these treatment strategies benefit patients with localized prostate cancer, their efficacy is restricted when prostate cancer becomes highly metastatic, spreading to other parts of the body, especially to the bone^14^. Hence, finding new molecular targets to diagnose and treat metastatic prostate cancer is urgently needed. However, the development of effective therapies for this advanced prostate cancer still remains a major clinical challenge. The process of tumor development and metastasis is multifaceted, and involves misregulation of both proto-oncogenes and tumor suppressor genes^11,15,16^. Also, functional inactivation of growth-inhibitory factors can lead to uncontrolled cell cycling and aberrant cell growth in those pathogenic processes^11,15^. Accordingly, the identification of specific molecular alterations that underlie the pathogenesis of prostate cancer metastasis may hold promise for predicting the clinical course and aggressiveness of newly diagnosed cases.

Anoctamin1 (ANO1) is a calcium-activated chloride channel (CaCC) that is widely expressed in a variety of human carcinomas, including head and neck squamous cell carcinoma, esophageal squamous cell carcinoma, oral cancer, breast cancer, and prostate cancer^17–22^. Related to the current report, ANO1 has been proposed to play a role in regulating the proliferation of prostate cancer cells and tumorigenesis in prostate cancer models. Interestingly, however, high levels of ANO1 expression were detected only in some prostate cancer cell lines such as PC3, and analysis of other prostate cancer cell lines including LNCaP and DU145 cells exhibited low levels of ANO1 expression^23^. These observations strongly suggest that there are different layers of signaling pathways to modulate ANO1 expression in those prostate cancer cell lines. In agreement with this idea, several previous studies have reported that ANO1 expression is regulated by epigenetic mechanisms involving promoter DNA methylation in head and neck squamous cell carcinomas and salivary glands^24,25^. Nevertheless, the exact mechanisms underlying the differential expression of ANO1 in LNCaP, DU145, and PC3 prostate cancer cells have not been well elucidated.

In this study, we investigated the expression pattern and oncogenic function of ANO1 using three prostate cancer cell lines with varying degrees of metastatic potential. Our results indicate a distinct role for DNA methylation in controlling the magnitude of ANO1 expression at the transcription level in the LNCap low-metastatic, DU145 moderately-metastatic, and PC3 highly-metastatic prostate cells. Using the DNA methylation inhibitor 5-Aza-CdR, we further demonstrate that DNA methylation attenuates migration and invasion of LNCaP and DU145 cells by suppressing ANO1 expression. Additionally, induction of ANO1 expression by 5-Aza-CdR treatment increased cancer characteristics, but silencing of ANO1 expression by RNAi attenuated the treatment effects, suggesting 5-Aza-CdR-induced metastatic potential of LNCaP and DU145 cells was generated through its major impact on ANO1 expression.

## Material and methods

### Cell culture

Human prostate cancer cell lines LNCaP, DU145, and PC3 were purchased from the ATCC (Manassas, VA, USA) and LNCaP or DU145 and PC3 cells were used throughout the study. LNCaP or DU145 and PC3 cells were cultured in RPM1-1640 medium or Dulbecco’s modified Eagle’s medium (DMEM) supplemented with 10% v/v inactivated fetal bovine serum (FBS) and 100 U/ml penicillin + 100 µg/ml streptomycin. The cell lines were incubated at 37 °C in a humidified atmosphere of 95% air and 5% CO_2_.

### RT-qPCR

LNCaP, DU145, and PC3 cells were treated with 10 μM 5-Aza-CdR for 1, 2, 3 and 4 days, and the medium containing 5-Aza-CdR was changed daily. Total RNA was isolated from untreated or 5-Aza-CdR-treated cells using the RNeasy Mini Kit (Qiagen, Hilden, Germany) according to the manufacturer’s instruction. cDNA was synthesized from 2 μg of total RNA using first-strand cDNA using the SuperScript III First-Strand System Kit (Thermo Fisher Scientific, Waltham, MA, USA). The real-time RT-qPCR was performed with SYBR Green Real-time PCR Master Mixes (Thermo Fisher Scientific, Waltham, MA, USA) using Agilent Aria 1.71 software according to the manufacturer’s protocol. The primers used for RT-qPCR are listed in Supplementary Table S1. All RT-qPCR reactions were run in triplicate, and results were normalized to β-actin mRNA levels.

### Western blot analysis

The harvested LNCap, DU145, and PC3 cells were washed with phosphate buffered saline (PBS) and dissolved in lysis buffer. The protein samples were separated using 10% SDS-PAGE and transferred to a 0.45 µM nitrocellulose membrane. After blocking with 5% non-fat dry milk (Rockland), the membrane was incubated at 4 °C with a primary antibody and washed with TBST. The membrane was washed with TBST, incubated at room temperature with a secondary antibody for 1 h, and visualized using an ECL reagent (Thermo Fisher Scientific, San Diego, CA, USA). All experiments were performed in triplicate. Antibodies used in this study are as follows: ANO1 antibody (1:1000) from Santa Cruz Biotechnology, Santa Cruz, CA, USA; ANO8 antibody (1:1000) from Thermo Fisher Scientific, San Diego, CA, USA; ANO10 antibody (1:2000) from Thermo Fisher Scientific, San Diego, CA, USA; HRP-conjugated anti-rabbit antibody (1:5000) from Santa Cruz Biotechnology, Santa Cruz, CA, USA.

### Immunofluorescence

LNCap, DU145, and PC3 cells were grown on cell culture slides (Thermo Fisher Scientific, San Diego, CA, USA) and treated with 10 μM 5-Aza-CdR (Sigma Aldrich, St. Louis, MO, USA) for 3 days. Then, these cells were fixed in 4% paraformaldehyde, washed with PBS, blocked with 10% donkey serum, and incubated overnight with an anti-human ANO1 primary antibody (1:200; Santa Cruz Biotechnology, Santa Cruz, CA, USA) at 4 °C. After washing with PBS, the samples were incubated with an Alexa Fluor^®^ 488 donkey anti-mouse IgG secondary antibody (1:200) and Alexa Fluor^®^ 594 donkey anti-mouse IgG secondary antibody (1:200) for 1 h at room temperature. The slides were mounted with Vectashield H-1200 including DAPI (Vector Laboratories, Burlingame, CA, USA) and visualized by a LSM700 Confocal Laser Scanning Confocal Microscope (Carl Zeiss, Jena, Germany). All experiments were performed in triplicate.

### Intracellular Cl^-^ ion flux monitoring

Intracellular Cl^-^ ion was monitored as described previously^25^. Briefly, LNCap, DU145, and PC3 cells were harvested and loaded with 10 mM MQAE in RPM1-1640 or DMEM for 30 min at 37 °C. The cells were washed with HBSS and attached to a cover glass coated by Cell-Tak (Corning Inc, Corning Inc., NY, USA). The cover-glass formed the bottom of the experimental chamber. Fluorescence was monitored by using a LSM700 Confocal Laser Scanning Microscope system, fitted with a filter cube No. 49 (Ex/Em = 365/445, Zeiss, Oberkochen, Germany). After baseline intensity calibration, the cells were treated with 10 µM N-aroylaminothiazole “activators” (Eact), a specific agonist for ANO1, and fluorescence variations were measured for a further 300 s at 5 s intervals. All experiments were performed in triplicate.

### Methylation-specific PCR

For methylation-specific PCR (MSP), genomic DNA was extracted using the QIAamp DNA Blood Mini Kit (Qiagen, Valencia, CA, USA) from LNCap, DU145, and PC3 cells after mock or 5-Aza-CdR treatment, and subjected to bisulfite conversion with the EpiTect Bisulfite Kit (Qiagen, Valencia, CA, USA) as described previously^25–27^. Subsequently, the bisulfite-treated genomic DNA was amplified using methylation-specific or unmethylation-specific primers to probe the ANO1 CpG islands as follows: M forward 5′-TTTTAAGGTAAAGGCGGGTC-3′ and M reverse 5′-CTCGATACGAAAAACGCCTA-3′; U forward 5′-TATTTTTAAGGTAAAGGTGGGTT-3′ and U reverse 5′-CTCAATACAAAAAACACCTAAAC-3′. The PCR products were separated by 1% agarose gel electrophoresis and visualized by ethidium bromide staining. All experiments were performed in triplicate.

### Bisulfite sequencing

Bisulfite conversion of genomic DNA was performed using the EpiTect Bisulfite Kit (Qiagen, Valencia, CA, USA) according to the manufacturer’s instructions. The modified genomic DNA was amplified with bisulfite-conversion-based primer pairs (forward 5′-AAAAATAAAATTTGGAGGGGTT-3′ and reverse 5′-CCCTAACTACCCCAACAAATAC-3′) that were designed by the Methyl Primer Express software (ver. 1.0). The PCR reactions were performed as follows: 94 °C for 5 min; 35 cycles of 94 °C for 45 s and 55 °C for 45 s; and a final cycle of 72 °C for 45 s at the end. The PCR products were purified with a Gel Extraction Kit (Qiagen, Valencia, CA, USA) and ligated into the pGEM-T easy vector (Promega, Madison, WI, USA). Five clones from each group were selected for bisulfite sequencing analysis following the previously described procedure^25–27^. All experiments were repeated at least three times.

### GEO dataset analysis and PPI network (STRING)

To assess differential expression of anoctamin family in LNCap, DU145 and PC3 cells, human gene expression arrays for prostate cancer cell lines were downloaded from the NCBI Gene Expression Omnibus (GEO) repository which is accessible at https://www.ncbi.nlm.nih.gov/geo. The expression profiling dataset GSE211721 consisted of two LNCap, two DU145, and two PC3 samples^28^. Differentially expressed genes were selected and analyzed using the Gene Specific Algorithm from Partek^®^ Flow^®^ software (Partek Inc., St. Louis, MO, USA). The predicted protein–protein interactions are generated by using the STRING database (http://string-db.org), which is a web-based tool for protein–protein interaction networks and functional enrichment analysis between interacting genes and proteins, as described previously^27^.

### Single cell motility study (Time-lapse imaging)

For single cell motility assays, mock-depleted control and ANO1-depleted LNCap, DU145, and PC3 cells were incubated at low confluence (4 × 10^4^ cells/cm^2^) for 3 days in the presence or absence of 5-Aza-CdR. After cell adhesion to the bottom of the dishes, cell motility was monitored every 30 min for 24 h by a JuLI™ Stage automated cell imaging system with a 4 × objective as described previously^27^. To maintain live cells, time-lapse imaging cells were kept in a humidified atmosphere containing 5% CO_2_ at 37 °C. Ten cells in each plate were randomly chosen for analysis and tracked using the ImageJ Manual Tracking plug-in. Chemotaxis and migration tool (Ibidi, Fitchburg, WI, USA), which is a software tool for data analysis, was used to quantitate and visualize data. The velocity of cells movement was represented as µm/min. All experiment were repeated at least three times.

### Cell viability assay

LNCaP, DU145, and PC3 cells were seeded in 96-well plates at a density of 0.5 × 10^4^ and treated with 5-Aza-CdR (0–40 µm) for 4 days. Their viability was then assessed by using the MTT (3-(4,5-dimethylthyazol-2-yl)-2,5-diphenyl-tetrazolium bromide) cell growth assay kit (Sigma Aldrich, St. Louis, MO, USA) as detailed previously^25–27^.

### Matrigel invasion assay

For cell invasion assays, mock-depleted control and ANO1-depleted LNCap, DU145, and PC3 cells were harvested after treating with 5-Aza-CdR for 3 days, suspended in culture medium containing 5% FBS, and plated into the pre-coated upper chamber with Matrigel (BD Biosciences, Franklin Lakes, NJ, USA) as previously described in detail^27^. Then, cells were allowed to invade toward 10% FBS in the lower chamber for 24 h in a humidified atmosphere containing 5% CO_2_ at 37 °C. Invaded cells on the underside of the transwell filters were fixed with 4% formaldehyde for 10 min and stained with 1% crystal violet for 1 h. After washing the filters with PBS, invaded cells were photographed and counted. All experiments were repeated independently three times.

### Xenograft animal model for bone metastasis

Athymic nude mice [(Crl:NU(NCr)‐Foxn1nu] were obtained from Charles River (Wilmington, MA, USA) and housed under specific pathogen-free conditions. To examine the bone metastatic effects of ANO1, we used four different groups as followings: Mock-treated LNCap, 5-Aza-CdR-treated LNCap, ANO1-expressed LNCap, and PC3 cells. These cells (1 × 10^6^ cells/femur) were respectively implanted into the distal femur of 6-week-old male mice (n = 5). The progression of bone metastasis was monitored using an in vivo imaging system (IVIS) detecting bioluminescence 1 and 30 days after implantation. At the end of the experiment, mice were euthanized, and the femur and tibial bone were excised to evaluate the bone metastatic capacity of the injected cells by bioluminescence imaging. All mouse experiments were performed according to protocols approved by Animal Care and Use Committee of University of Southern California (Approval Number: 11673-CR012) and were conducted according to the guidelines of the Care and Use of Laboratory Animals published by the US National Institutes of Health. We complied with the ARRIVE guideline 2.0 for this reporting of animal experiment conducted in the present study.

### Statistical analysis

Statistical analysis was conducted using GraphPad Prism 9 software (ver. 9.0.0). The data were compared using one- or two-way analysis of variance (ANOVA) with post-hoc Tukey or Bonferroni test for multiple comparisons. All data were presented as mean ± standard error of the mean (SEM). A *P*-value of 0.05 was considered statistically significant.

## Results

### Restoration of ANO1 expression following 5-Aza-CdR treatment

**Figure 1:**
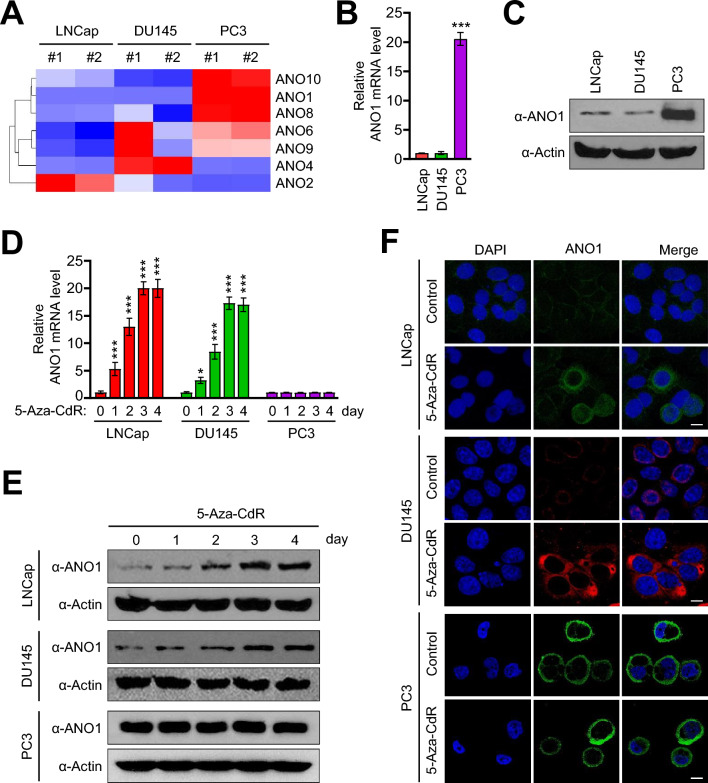
5-Aza-CdR treatment effects on ANO1 expression in prostate cancer cells. (**A**) Heatmap showing mRNA expression profiles of anoctamin family in LNCap, DU145, and PC3 cells. (**B**) Total RNA samples were prepared from LNCap, DU145, and PC3 cells, and ANO1 mRNA levels were analyzed by RT-qPCR with primers listed in Supplementary Table S1. All transcription levels were normalized to that of GAPDH. Data represent the mean ± SD of three independent experiments in triplicate; ***P < 0.001. (**C**) Whole cell lysates were prepared from LNCap, DU145, and PC3 cells and analyzed by Western blotting with ANO1 antibody. Actin served as a control for equal protein loading. Shown are the representative results of three independent Western blot experiments. (**D**) LNCap, DU145, and PC3 cells were grown in the absence or presence of 10 µM 5-Aza-CdR for 0, 1, 2, 3, or 4 days. Then, RNA samples were prepared and analyzed by RT-qPCR as in (**B**). Data represents the mean ± SD of three independent experiments in triplicate; *P < 0.05, ***P < 0.001 versus 0 day. (**E**) Western blot analysis was performed as in (**D**), but after treating LNCap, DU145 and PC3 cells with 5-Aza-CdR over a period of 0, 1, 2, 3, or 4 days. Actin served as a control for equal protein loading. Shown are the representative results of at least three independent Western blot experiments. (**F**) LNCap, DU145, and PC3 cells were immunostained with ANO1 antibody 3 days after mock-treatment or 5-Aza-CdR treatment. Scale bars correspond to 15 μm. Representative images of stained cells are shown.

Although calcium-activated chloride channel anoctamin (ANO) family members have been reported to be highly expressed in prostate cancer, their impacts on the metastatic potential of prostate cancers are largely unknown. To test the possibility that ANO family influences the pathogenesis of metastatic prostate cancer, we first downloaded the dataset of GSE211721 from Gene Expression Omnibus (GEO) database and analyzed the expression of ANO family members in low metastatic (LNCaP), moderately metastatic (DU145), and advanced metastatic (PC3) human prostate cancer cell lines. In this initial characterization, we found that ANO family members have distinct expression patterns in LNCaP, DU145, and PC3 prostate cancer cell lines. One of the key observations made in our analysis was much lower expression of ANO1, ANO8 and ANO10 in LNCap low-metastatic and DU145 moderately-metastatic cells when compared to PC3 highly-metastatic prostate cancer cells (Fig. 1A). Consistent with these observations, RT-qPCR and Western blot analyses showed that ANO1, ANO8, and ANO10 are minimally expressed in LNCap and DU145 cells but highly expressed in PC3 cells (Fig. 1B,C and Supplementary Fig. S1A,B).

Since DNA methylation has been reported as a regulatory mechanism for the expression of ANO family members^25^, the elevated expression of ANO1, ANO8, and ANO10 in PC3 cells (Fig. 1) encourages the possibility that DNA methylation could serve as a key step to control their expression. Also, considering that DNA methylation mainly affects gene expression at the level of transcription, it was reasonable to speculate that transcription is the primary process through which the expression of ANO1, ANO8, and ANO10 is regulated in LNCaP, DU145, and PC3 prostate cancers. In good agreement with this idea, our RT-qPCR analysis detected approximately 20-fold increase in ANO1 mRNA levels after exposing LNCap and DU145 cells to 10 µM 5-Aza-CdR over a period of four days (Fig. 1D). We also observed significant accumulation of ANO1 protein after treatment with 10 µM 5-Aza-CdR when conducted Western blotting. Notably, however, ANO8 and ANO10 expression remained unaffected at both mRNA and protein levels following 5-Aza-CdR treatment (Supplementary Fig. S1C) indicating that DNA methylation is not a regulatory mechanism employed for these genes. Moreover, the observed changes in ANO1 mRNA and protein levels were not due to increased cell death, because the viability of LNCap, DU145, and PC3 cells was not significantly affected 4 days after exposing to the DNMT inhibitor 5-Aza-CdR at any concentration up to 10 µM (Fig. 1D,E and Supplementary Fig. S2). To further support the results from RT-qPCR and Western blot analyses, LNCap and DU145 cells were subjected to immunofluorescence staining with anti-ANO1 antibody after 5-Aza-CdR treatment. As can be seen in Fig. 1F, there was a significant increase in ANO1 signals on the plasma membrane in 5-Aza-CdR-treated LNCap and DU145 cells, again indicating that 5-Aza-CdR treatment is sufficient to induce ANO1 expression in prostate cancer cells. Unlike LNCaP and DU145 cells, no changes were detected when our assays were repeated with 5-Aza-CdR-treated PC3 cells. Considered together, these initial observations suggest that DNA methylation plays a critical role in down-regulating ANO1 expression and that 5-Aza-CdR treatment efficiently attenuates DNA-methylation-induced ANO1 silencing in prostate cancer cells with low metastatic potential.

### 5-Aza-CdR-induced alteration in ANO1-locus DNA methylation and ANO1 function

**Figure 2:**
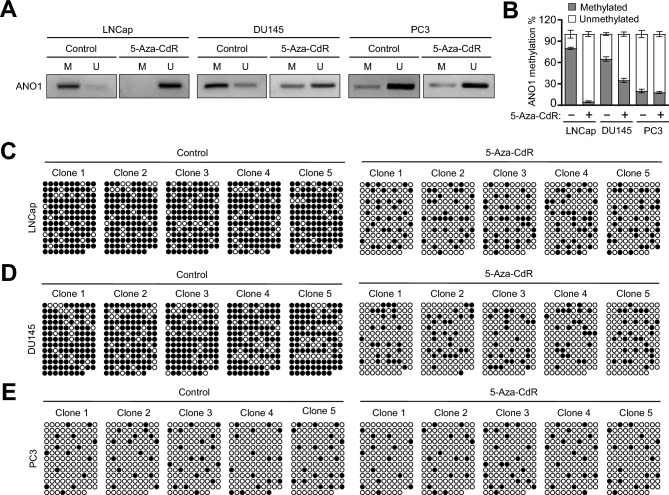
5-Aza-CdR treatment effects on DNA methylation at the ANO1 CpG island locus. (**A**) LNCap, DU145, and PC3 cells were mock-treated or 5-Aza-CdR-treated for 3 days, and methylation-specific PCR (MSP) was carried out with sodium bisulfite-modified genomic DNA. M and U indicate PCR products of the methylated and unmethylated ANO1 CpG islands, respectively. (**B**) The intensity of bands in (**A**) was quantified by measuring the optical density. Data represent the mean ± SD for the percentage ratio between methylation-specific and unmethylation-specific bands (n = 3). (**C**–**E**) Bisulfite sequencing was performed to analyze five separate clones of ANO1 CpG island in mock-treated or 5-Aza-CdR-treated LNCap (**C**), DU145 (**D**), and PC3 (**E**) cells. White circles indicate unmethylated CpG sites while black circles indicate methylated CpG sites.

As an extension of the above-described studies demonstrating the stimulatory effects of 5-Aza-CdR treatment on ANO1 expression, we next wanted to study how 5-Aza-CdR treatment influences DNA methylation occurring in ANO1 gene. Toward this end, we first analyzed ANO1 gene for the presence of CpG islands using the MethPrimer software^29^ and identified a CpG island (CGI) containing 128 CpGs located in the coding region between positions 70,078,169 and 70,079,120 (Supplementary Fig. S3). We then examined the impact of 5-Aza-CdR treatment on DNA methylation status in this CGI by conducting methylation-specific PCR with a pair of primers specific for the methylated (M) and unmethylated (U) sequences. A noteworthy observation emerged from our results was that 5-Aza-CdR treatment of LNCap and DU145 prostate cancer cells severely diminished the intensity of methylation-specific bands but increased unmethylation-specific amplicon in the region of the CpG island (Fig. 2A,B). On the contrary, ANO1 in PC3 cells represented a highly unmethylated CpG island and showed no changes in the intensity of methylation-specific bands after 5-Aza-CdR treatment (Fig. 2A,B). As a more direct approach toward identifying conversion of DNA methylation status at CpG dinucleotides, DNA bisulfite sequencing was performed on the ANO1 coding region including 128 CpG sites in LNCap, DU145, and PC3 cells (Supplementary Fig. S3). In mock-treated LNCap and DU145 cells, the CpG sites in the coding region of ANO1 were highly methylated, with approximately ~ 80% of CpG sites containing 5-methylcytosine (Fig. 2C,D). The 5-Aza-CdR treatment generated an approximate 60% reduction in the observed methylation level at CpG sites. (Fig. 2C,D and Supplementary Fig. S4). Conversely, mock-treated PC3 cells displayed CpG methylation levels below 20%, and the extent of observed methylation remained almost unaffected by 5-Aza-CdR treatment (Fig. 2E and Supplementary S4). These results indicate that 5-Aza-CdR treatment induces demethylation of CpG sites on ANO1 coding region in low-metastatic LNCap and DU145 prostate cancer cells and has no to very limited effects in highly-metastatic PC3 prostate cancer cells.

**Figure 3:**
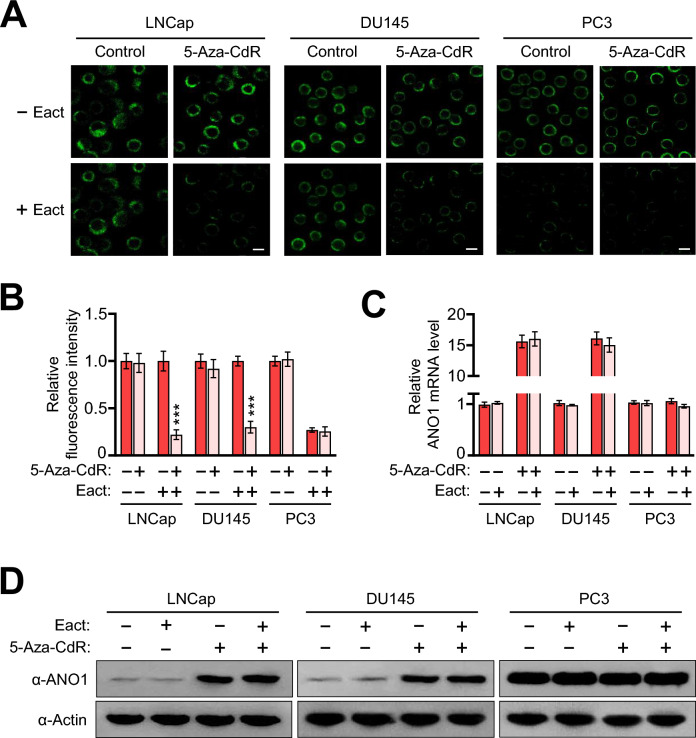
5-Aza-CdR treatment effects on ANO1 function in prostate cancer cells. (**A**) LNCap, DU145, and PC3 cells were treated with 10 µM Eact and/or 10 µM 5-Aza-CdR as indicated, and changes in intracellular Cl^-^ were monitored using (6-Methoxyquinolinio) acetic acid ethyl ester bromide [MQAE] for 300 s. Images are representative of three independent experiments. Scale bars correspond to 50 μm. (**B**) The mean changes in fluorescence were measured from the cells used in (**A**). Data are represented as mean ± SEM of three independent experiments in triplicate; ***P < 0.001. (**C**) After treating LNCap, DU145, and PC3 cells as in (**A**), total RNA was isolated and analyzed by RT-qPCR using primers listed in Supplementary Table S1. All transcription levels were normalized to that of GAPDH. Data represent the mean ± SD of three independent experiments in triplicate. (**D**) LNCap, DU145, and PC3 cells were treated as described in (**A**), and whole cells lysates were prepared and analyzed by Western blotting with ANO1 antibody. Actin antibody was used as a control. Data are representative of three independent experiments.

To extend the above observations and further evaluate the effects of 5-Aza-CdR treatment, it was important to explore whether the ANO1 expression by 5-Aza-CdR-induced DNA demethylation is functional in prostate cancer cells. In approaching this question, we decided to analyze ANO1-mediated flow of chloride ions (Cl^-^) using an intracellular fluorescence Cl^-^ sensor, (6-Methoxyquinolinio) acetic acid ethyl ester bromide (MQAE). MQAE is a fluorescent indicator and used to monitor changes in intracellular Cl^-^ concentration and activity. To stimulate Cl^-^ flow through ANO1, we treated LNCap, DU145, PC3 prostate cancer cells with N-aroylaminothiazole (Eact) which is a specific agonist for ANO1. As shown in Fig. 3A and summarized in Fig. 3B, a significant decrease in MQAE fluorescence, indicative of higher change in intracellular Cl^-^ concentration, was observed with 5-Aza-CdR-treated LNCap and DU145 cells, as well as in 5-Aza-CdR-treated PC3 cells, following their 5 min exposure to Eact. Conversely, when assays were repeated in the absence of 5-Aza-CdR, Eact failed to generate any significant changes in intracellular Cl^-^ concentrations in LNCap, and DU145 cells (Fig. 3A,B). Because ANO1 is overexpressed and functional in PC3 cells, we also observed some distinct changes in response to Eact treatment (Fig. 3A,B). Although these results showed Cl^-^ movement through membrane-localized ANO1 in the presence of 5-Aza-CdR, we cannot exclude the possible effects of Eact on ANO1 expression. However, Eact failed to generate any effects on ANO1 mRNA and protein levels in LNCap and DU145 cells (Fig. 3C,D), and this observation strongly suggests that Eact solely acts in producing sustained Cl^-^ currents. Collectively all these results are supportive of the view that DNA demethylation is a critical mechanism for inducing ANO1 expression and functional activation on the membrane of LNCap and DU145 prostate cancer cells.

### ANO1-mediated regulation of pro-metastatic genes

**Figure 4:**
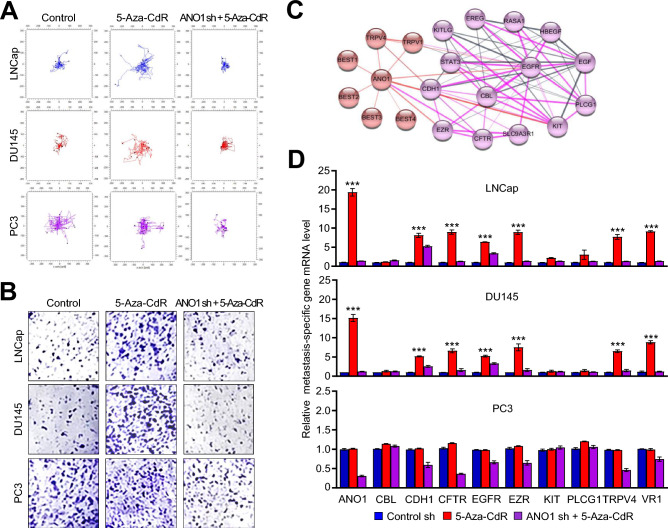
A positive correlation of ANO1 expression with metastatic characteristics in 5-Aza-CdR-treated cells. (**A**) LNCap, DU145, and PC3 cells were mock-depleted or depleted of ANO1, and then incubated in the presence or absence of 10 µM 5-Aza-CdR. Three days after 5-Aza-CdR treatment, the single cell motility was monitored by a time-laps imaging system for 24 h. Shown are the representative time-lapse images for each experimental condition. (**B**) The Matrigel invasion assay was performed with LNCap, DU145, and PC3 cells used in (**A**). The invaded cells were stained with 1% crystal violet and visualized under a light microscope. Representative images are shown. (**C**) The protein–protein interaction (PPI) network of ANO1 protein was analyzed using the search tool for retrieval of interacting genes (STRING) database (https://string-db.org/). The graphics indicate a possible physical interaction between proteins. (**D**) After treating LNCap, DU145, and PC3 cells as in (**A**), total RNA samples were prepared and analyzed by RT-qPCR with primer listed in Supplementary Table S1. All transcription levels were normalized to that of GAPDH. Data are represented the means ± SD of three independent experiments in triplicate; ***P < 0.001 versus control sh.

Having identified the mechanistic basis for an aberrant expression of ANO1 in prostate cancer cells, we next directed our attention toward understanding its impacts on the metastatic potential of prostate cancer cells. Accordingly, we knocked down ANO1 in LNCap, DU145 and PC3 cells using a lentiviral shRNA infection system which allows a prolonged depletion for ANO1 and thus our study under identical conditions. As shown in Supplementary Fig. S5, the stable transfection of ANO1 shRNA efficiently silenced the expression of ANO1 expression in the cancer cells. Using these ANO1-depleted cells, we examined whether the reactivation of ANO1 in response to 5-Aza-CdR treatment could influence the migratory and invasive abilities of prostate cancer cells, which are considered as critical determinants for metastatic cell dissemination. When time-lapse imaging was employed to evaluate the motility of 10 cancer cells every 30 min over a 24 h period, a statistically significant increase in their motility was observed after 5-Aza-CdR treatment of LNCap and Du145 cells. On the other hand, 5-Aza-CdR treatment resulted in no change in the motility characteristics of PC3 cells. Moreover, 5-Aza-CdR treatment was no longer capable of stimulating the migratory ability of LNCap and DU145 cells when ANO1 was depleted (Fig. 4A and Supplementary Fig. S6A). Consistent with expectations from these results, 5-Aza-CdR-treated LNCap and DU145 cells also exhibited an increased invasion capacity through Matrigel, compared with mock-treated control cells in our Matrigel invasion assays, (Fig. 4B and Supplementary Fig. S6B). The observed response to 5-Aza-CdR treatment is dependent on ANO1, because ANO1-depleted cells failed to fully recapitulate 5-Aza-CdR effects without showing much higher invasive capacity (Fig. 4B and Supplementary Fig. S6B).

As a way to provide additional evidence supporting the data presented above, we also performed the analysis of the protein–protein interaction (PPI) network using the search tool for retrieval of interacting genes (STRING) database (https://string-db.org/). This additional study revealed a close functional link of ANO1 with a group of metastasis-related proteins, as summarized in Fig. 4C. To valid this connection, we conducted RT-qPCR on mock-depleted control and ANO1-depleted LNCap and DU145 cells following treatment with 5-Aza-CdR. Expectedly, 5-Aza-CdR treatment considerably enhanced ANO1 expression and markedly activated gene networks related to metastatic signaling modules, including CDH1, CFTR, EGFR, EZR, TRPV4, and VR1 (Fig. 4D). Also, our examination of those six activated genes in ANO1-depleted cells clearly demonstrated that their activated expression in response to 5-Aza-CdR treatment is dependent on ANO1 (Fig. 4D). All these findings were further supported by the observation that advanced metastatic PC3 cells expressing high levels of ANO1 do not show much changes in migration and invasion capacity as well as six downstream gene expression upon 5-Aza-CdR treatment (Fig. 4 and Supplementary Fig. S6). Taken together, these data underscore the essential role for ANO1 in upregulating migration and invasion abilities of prostate cancer cells and support the concept that ANO1 is a necessary factor to upregulate genes encoding potential inducers of metastasis in prostate cancer cells.

### Stimulation of prostate *cancer*-induced bone metastasis by ANO1

**Figure 5:**
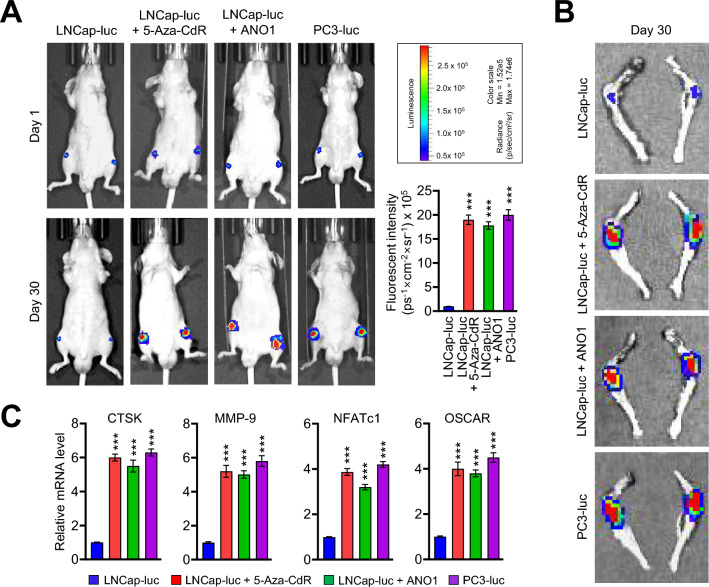
ANO1-dependent stimulation of prostate cancer cell bone metastasis by 5-Aza-CdR. (**A**) Xenografts were established by injecting mock-expressing control or ANO1-expressing LNCap and PC3 cells into the distal femur and proximal tibia of mice and treated with DMSO or 5-Aza-CdR for 30 days. Tumor growth was monitored in LNCap and PC3 xenografts by detecting bioluminescence with an in vivo imaging system (IVIS) after 1 and 30 days of treatments. The bioluminescence intensity was quantified using regions of interest (ROIs) of equivalent-sized areas at the indicated time. Data represent the mean ± SD (n = 5); ***P < 0.001 versus LNCap-luc. (**B**) After treating mice bearing LNCap and PC3 xenograft as in (**A**), mice were sacrificed, and the bones of the hindlimb were excised from the distal legs. The bone metastatic tumor cells on the hindlimb were visualized by detecting bioluminescence with an IVIS. (**C**) Total RNA samples were isolated from tibial bone in mice for 30 days after DMSO or 5-Aza-CdR treatment. RT-qPCR was performed using primers listed in Supplementary Table S1. All transcription levels were normalized to that of GAPDH. Data represent the mean ± SD of three independent experiments in triplicate; ***P < 0.001 versus LNCap-luc.

The results of the above in vitro experiments argue persuasively that ANO1 plays an important role in controlling the metastatic potential of prostate cancer cells. However it is not clear whether the observed effects of ANO1 truly reflect its physiological activities during the spread of prostate cancer to other tissues. In addressing this question, we decided to use an animal model of bone metastasis, since more than 70% of prostate cancer patients have detectable bone metastases and since bone metastases are a major cause of prostate cancer-related mortality. To this end, we implanted into the distal end of both femurs of mice low-metastatic LNCap (LNCap-luc) and highly-metastatic PC3 (PC3-luc) cells stably expressing luciferase and checked their ability to spread to bone. When we monitored the development of bone metastasis with or without ANO1 expression and 5-Aza-CdR treatment using an in vivo imaging system (IVIS), a small hot spot of bioluminescence was observed at 1 day after implantation in all cases (Fig. 5A). However, at 30 days after cell implantation, 5-Aza-CdR treatment caused a significant enhancement of bone metastatic progression of LNCap cells (Fig. 5A,B, LNCap-luc + 5-Aza-CdR). Importantly, the fact that the ANO1 expression in LNCap cells facilitates migration ability to the tibial bone (Fig. 5A,B, LNCap-luc + ANO1) strongly argues that 5-Aza-CdR treatment promotes bone metastatic potential of LNCap cells through ANO1 reactivation.

A series of recent studies including ours reported that bone metastasis of prostate cancer enhances the expression of osteoclastogenic genes and increases the rate of bone resorption^30,31^. Therefore we also hypothesized that 5-Aza-CdR treatment and ANO1 expression will potentiate the expression of genes necessary for osteoclast differentiation. In fact, consistent with this idea, our RT-qPCR analyses demonstrated that 5-Aza-CdR treatment and ANO1 expression caused four- to sixfold increases in the expression of osteoclastogenic transcription factors CTSK, MMP-9, NFATc1 and OSCAR^26,30,31^. Meanwhile, a parallel experiment with highly-metastatic PC3 cells showed the high levels of those gene expression even in the absence of 5-Aza-CdR treatment (Fig. 5C). These results reinforce our conclusion that 5-Aza-CdR-induced ANO1 expression has positive impact on metastatic potential of prostate cancer cells and leads to osteolytic lesions by upregulating osteoclast differentiation at the metastatic bone lesion.

## Discussion

Epigenetic mechanisms controlling gene transcription in mammalian cells can be broadly classified into two main categories: DNA methylation and histone modification^3,32–34^. Notably, DNA methylation entails the addition of a methyl group at position C5 of cytosine residues within CpG islands and represents the most critical DNA modification process. Mounting evidence suggests that dysregulation of DNMT activities is associated with aberrant hypo- or hypermethylation and has profound effects on gene transcription. Along with these indications, recent investigations continue to unveil the pivotal role of aberrant DNA methylation and related epigenetic processes in cancer initiation, promotion, and progression^35–37^. Moreover, there are several reports that highlight a role of DNA methylation in regulating multiple facets of cancer metastatic process^4,32–34,38^. ANO1 is often highly amplified and expressed in prostate cancer cells, and is functionally correlated with invasive and metastatic potential of cells^21,39^. Despite the growing interest in its function as a proto-oncogene, not much is known about epigenetic pathways that control of ANO1 expression in prostate cancer cells. Interestingly, our initial characterization demonstrated that ANO1 is expressed at significantly lower levels in low metastatic LNCap and DU145 prostate cancer cells than in high metastatic PC3 prostate cancer cells. Considering that DNA methylation is a vital epigenetic mechanism that cells use for gene silencing, we hypothesized that DNA methylation could potentially attenuate the expression and thus function of ANO1 in prostate cancer cells. Our data support this hypothesis and demonstrate that treatment with DNMT inhibitor 5-Aza-CdR can reactivate ANO1 gene transcription in LNCap and DU145 cells. This study represents an extensive analysis of DNA methylation at the ANO1 gene locus and provides important insights into how ANO1 expression is epigenetically regulated in prostate cancer cells, extending our view on DNA methylation as a key determinant of prostate cancer development.

Since ANO1 is known to play a role in the metastatic cascade of prostate cancer^23^, a question raised from this finding is whether invasive and metastatic potentials of LNCap and DU145 cells are also influenced by 5-Aza-CdR treatment. In exploring this possibility, we found that transcriptional activation of ANO1 in 5-Aza-CdR-treated LNCap and DU145 cells is directly linked to the enhancement of metastatic hallmarks, such as mobility and invasiveness. These results raised another question of whether 5-Aza-CdR treatment also mediates transcriptional activation of genes encoding metastasis-promoting factors in LNCap and DU145 prostate cancer cells. In fact, the predicted influence is supported by the observation that several representative metastasis-related genes such as CDH1, CFTR, EGFR, EZR, TRPV4, and VR1 are activated in their transcription levels in response to 5-Aza-CdR treatment. Remarkably, however, the observed effects of 5-Aza-CdR treatment almost completely disappeared when ANO1 was depleted from LNCap and DU145 cells. This observation is intriguing because it supports the idea that ANO1 is an essential upstream regulator of metastasis-associated genes. Thus, it is tempting to speculate that 5-Aza-CdR can reduce their mRNA expression levels through exerting its influence on ANO1 transcription. Moreover, DNA demethylation may be considered as a crucial mechanism to regulate ANO1 expression and function in metastasis-associated transcriptional program in prostate cancer cells. Consistent with expectations from these results, our in vivo data underscore the role of ANO1 in increasing metastatic gene expression, cancer cell bone relocation, and osteoclastic bone lysis. Such a connection between ANO1 and bone metastasis has been demonstrated by previous studies exploring ANO1 as a regulator of lung metastatic potential of gastric cancer^40^. However, the mechanism behind the control of ANO1 expression in certain metastatic cancers has not been elucidated yet. Our data clearly implicate DNA methylation for ANO1 gene silencing and underscore the importance of this epigenetic process in modulating prostate cancer bone metastasis. Additionally and significantly, our studies using in vivo models also provided a new level of insight into the direct association between causative ANO1 expression and resulting phenotypic alterations with respect to prostate cancer bone metastasis. In this regard, ANO1 is a hub promoting metastatic potentials in prostate cancer cells and a promising target for novel therapeutic strategies for bone metastatic prostate cancer.

In summary, ANO1 is overexpressed in highly metastatic PC3 cells, but shows minimal expression in low metastatic LNCap and DU145 prostate cancer cells, indicating its possible role in regulating metastatic potential in prostate cancer. Adding support to the idea that ANO1 expression is epigenetically regulated, 5-Aza-CdR treatment generates the hypomethylation of ANO1 CpG islands and the active state of ANO1 gene expression in LNCap/DU145 prostate cancer cells and xenograft models. Subsequent reactivation of several downstream pro-metastatic genes leads to the initiation of prostate cancer bone metastasis as well as the development of osteolytic lesions. Based on these observations, together with findings from previous studies, epigenetic regulation of ANO1 expression can be viewed as a crucial process to modulate prostate cancer-derived bone metastasis.

### Supplementary Information

Supplementary Figures.

Supplementary Table 1.

## Data Availability

The data supporting the findings of this study are available within the paper and in the supplementary information file. Materials are available from the corresponding author, Woojin An, upon request.
