# Supplementary material for: Promoter hypermethylation as a novel regulator of ANO1 expression and function in prostate cancer bone metastasis
Source: Sci Rep. 2024 May 21;14:11595. doi: 10.1038/s41598-024-62478-1 (PMC11109272; doi:10.1038/s41598-024-62478-1)

**Supplementary Figures**


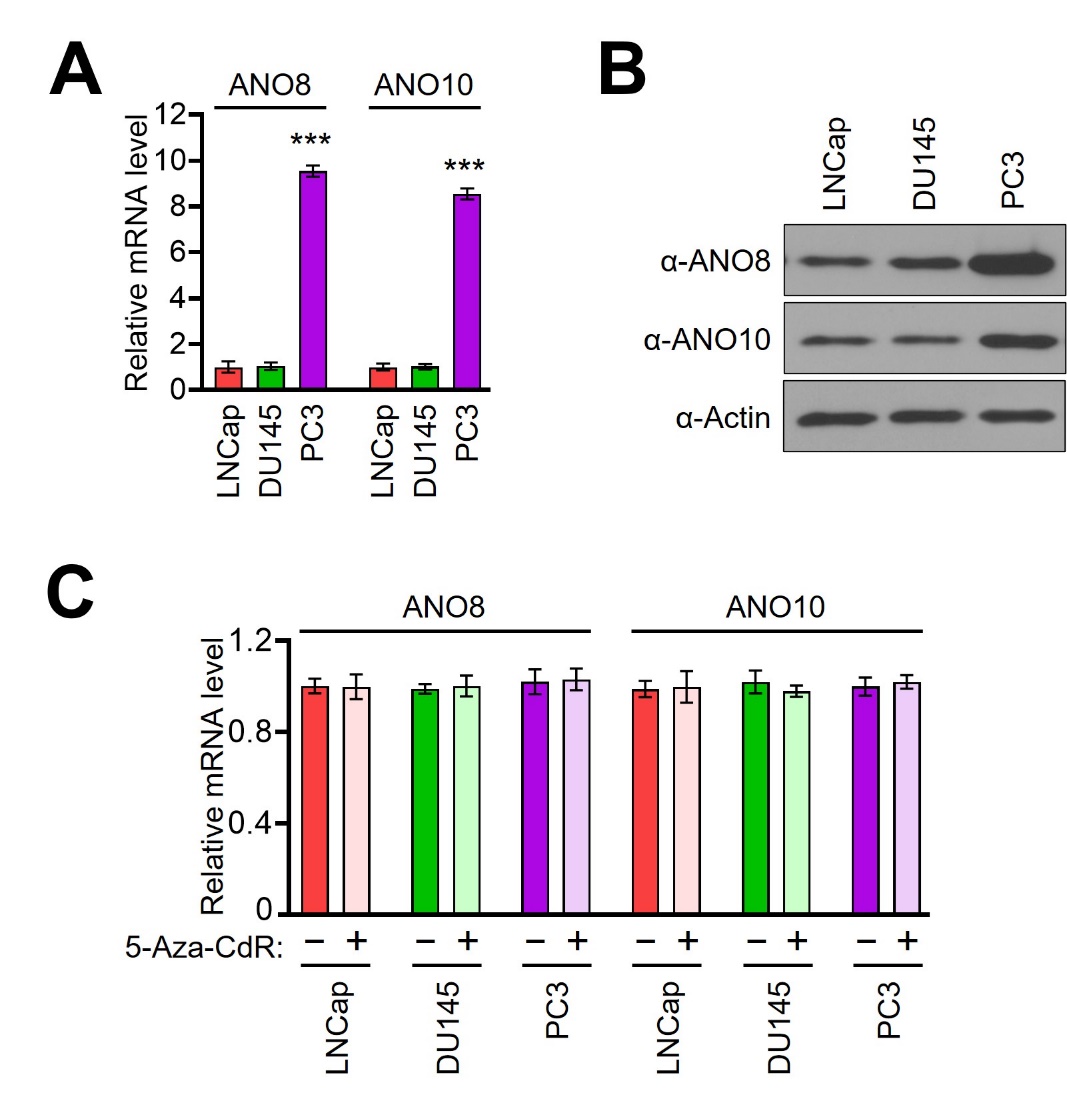


**Supplementary Figure 1. ANO8 and ANO10 expression levels in prostate cancer cells.**

(A) Total RNA was isolated in LNCap, DU145, and PC3 cells and analyzed by RT-qPCR using primers listed in Supplementary Table S1. All transcription levels were normalized to that of GAPDH. Data represent the mean ± SD of three independent experiments in triplicate; ***P < 0.001 versus LNCap.

(B) Whole cellular extracts were prepared from LNCap, DU145, and PC4 cells and analyzed by Western blot analysis with indicated antibodies. Data are representative of three independent experiments.

(C) After treating prostate cells with 5-Aza-CdR, total RNA was prepared and analyzed by RT-qPCR using primers listed in Supplementary Table S1. All transcription levels were normalized to that of GAPDH. Data represent the mean ± SD of three independent experiments in triplicate; ***P < 0.001 versus 0 day.

**
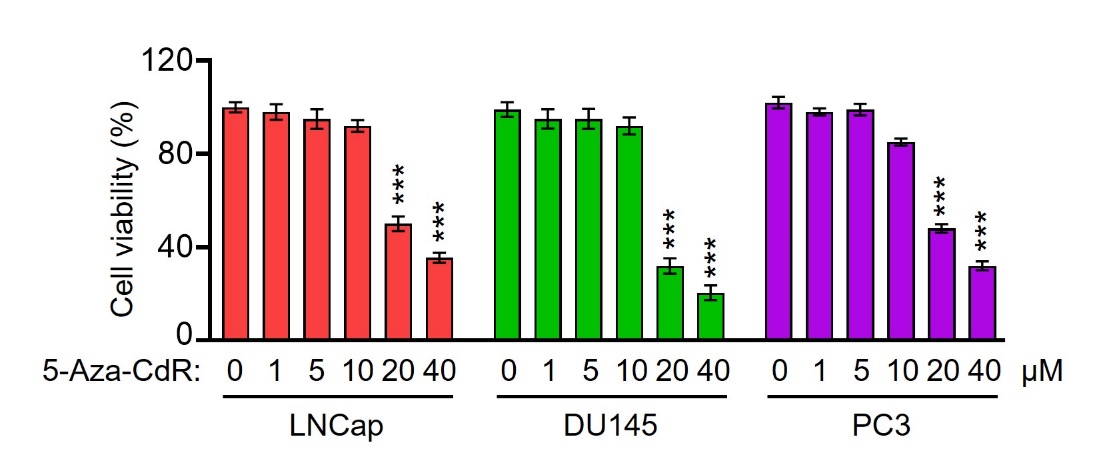
**

**Supplementary Figure 2.** **5-Aza-CdR treatment effects on prostate cancer cell growth.**

LNCap, DU145, and PC3 cells were treated with increasing concentrations of 5-Aza-CdR (0-40 µM) for 4 days, and their viability was evaluated by MTT assay. Data represent the mean ± SD of three independent experiments; ***P < 0.001 versus 0 µM.

**
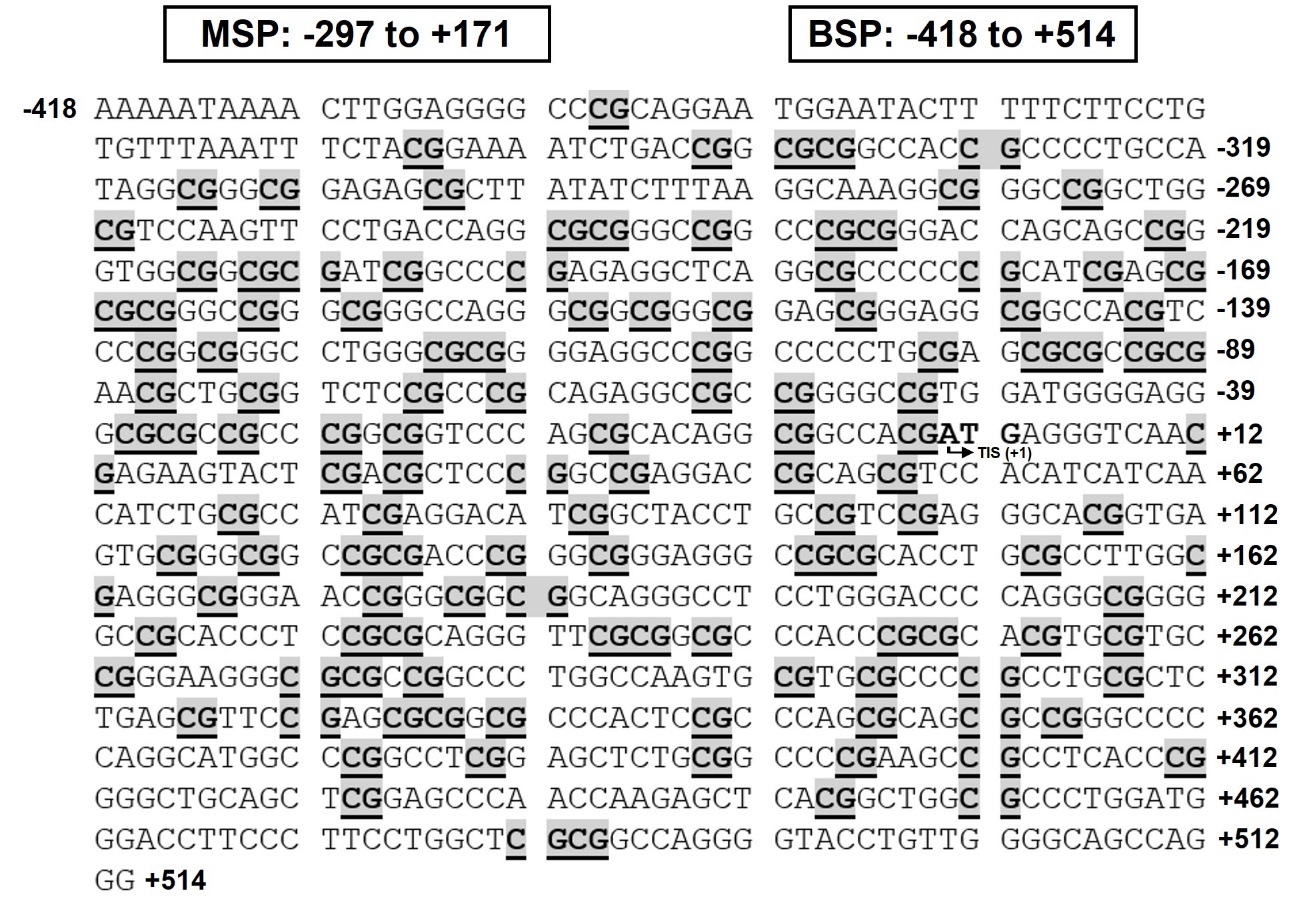
**

**Supplementary Figure 3. Sequence of the ANO1 CpG island locus.**

The ANO1 CpG island (NCBI accession: NC_000011, region: 70078169 to 70079120) contains 128 CpGs that are highlighted and underlined in bold.

**
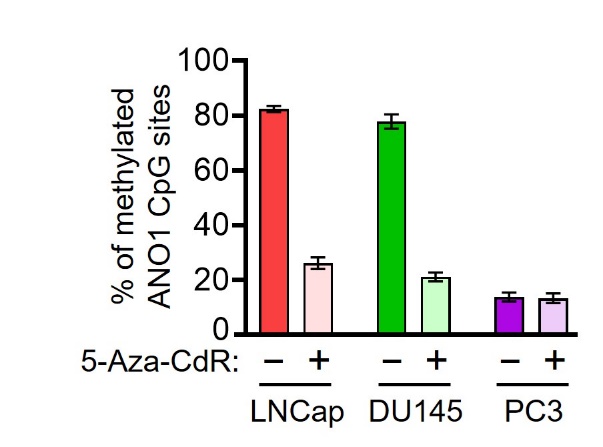
**

**Supplementary Figure 4. Status of methylated CpGs in ANO1 CpG island.**

The total numbers of methylated CpGs in Fig. 2C-E were counted and summarized. Data are represented as the mean ± SD for a percentage of the total number of CpGs (n = 5).

**
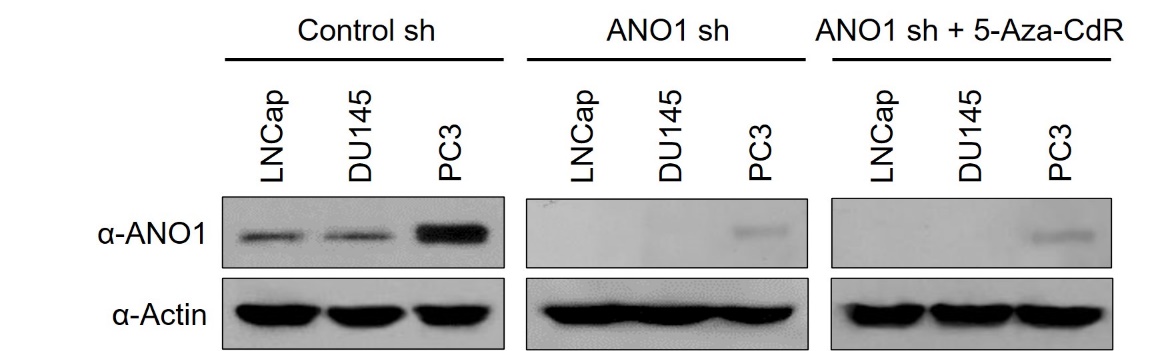
**

**Supplementary Figure 5. ANO1 knockdown and expression levels in prostate cancer cells.**

LNCap, DU145, and PC3 cells were mock-depleted or depleted of ANO1, and treated with 10 µM 5-Aza-CdR for 3 days. Whole cell lysates were prepared, and ANO1 levels were analyzed by Western blotting. Actin served as a control for equal protein loading. Data are representative of three independent experiments.

**
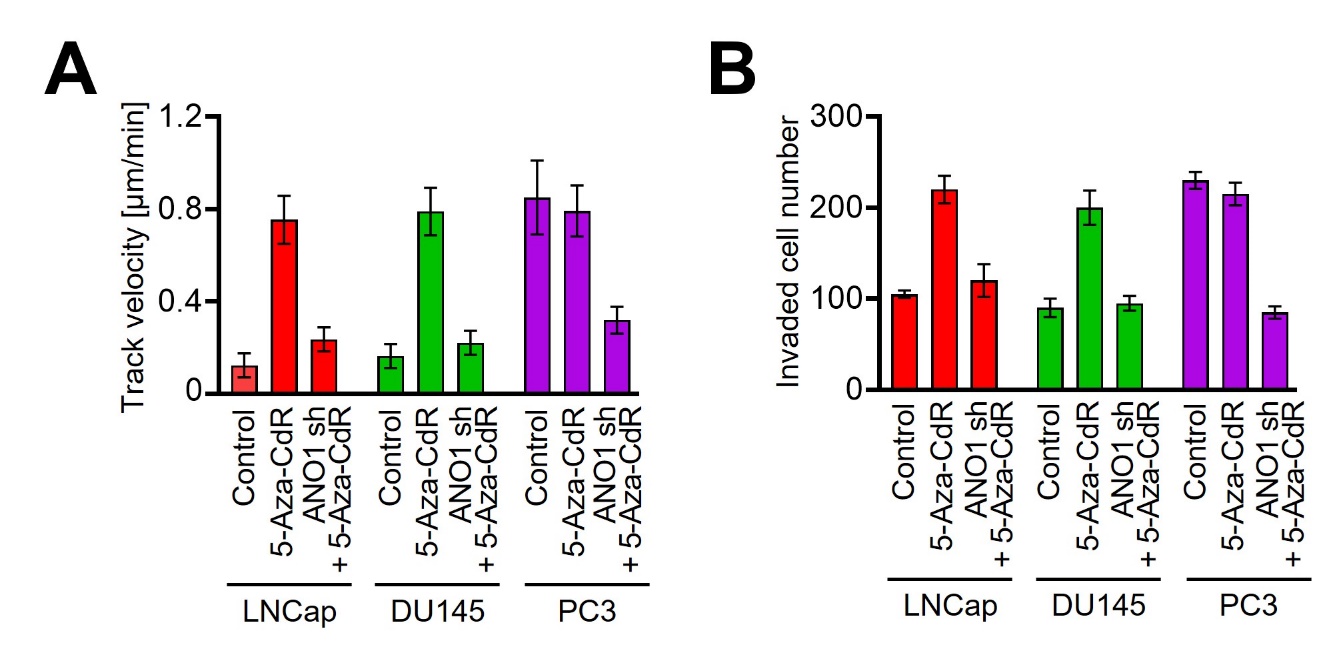
**

**Supplementary Figure 6. 5-Aza-CdR treatment effects on cell tracking and invasion.**

(A) The histogram displays a single cell track in Figure 4A, and the data are presented as the mean ± SD of the tracking of 10 cells.

(B) The average number of invaded cells in Figure 4B was quantified by ImageJ software (National Institutes of Health). Data represent the mean ± SD of three independent experiments in triplicate.

**The uncropped original image in Figure 1**

**1C**


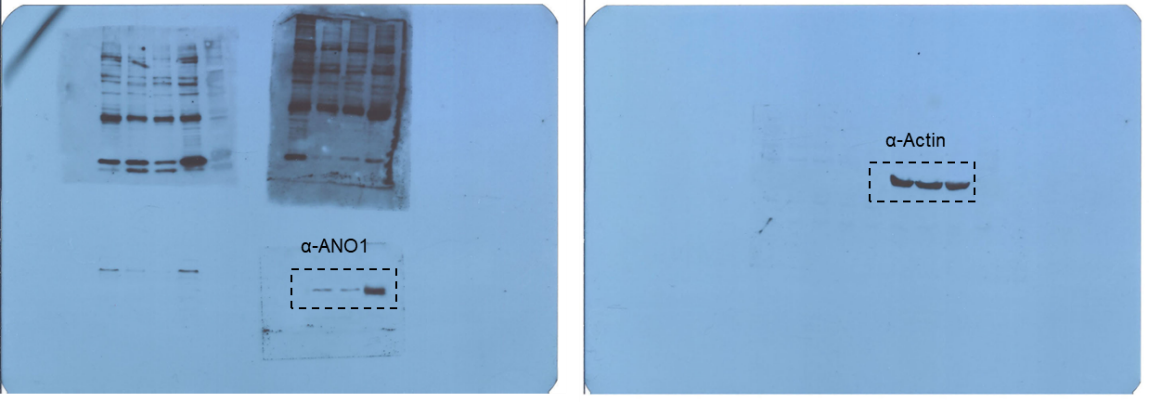


**1E**

**
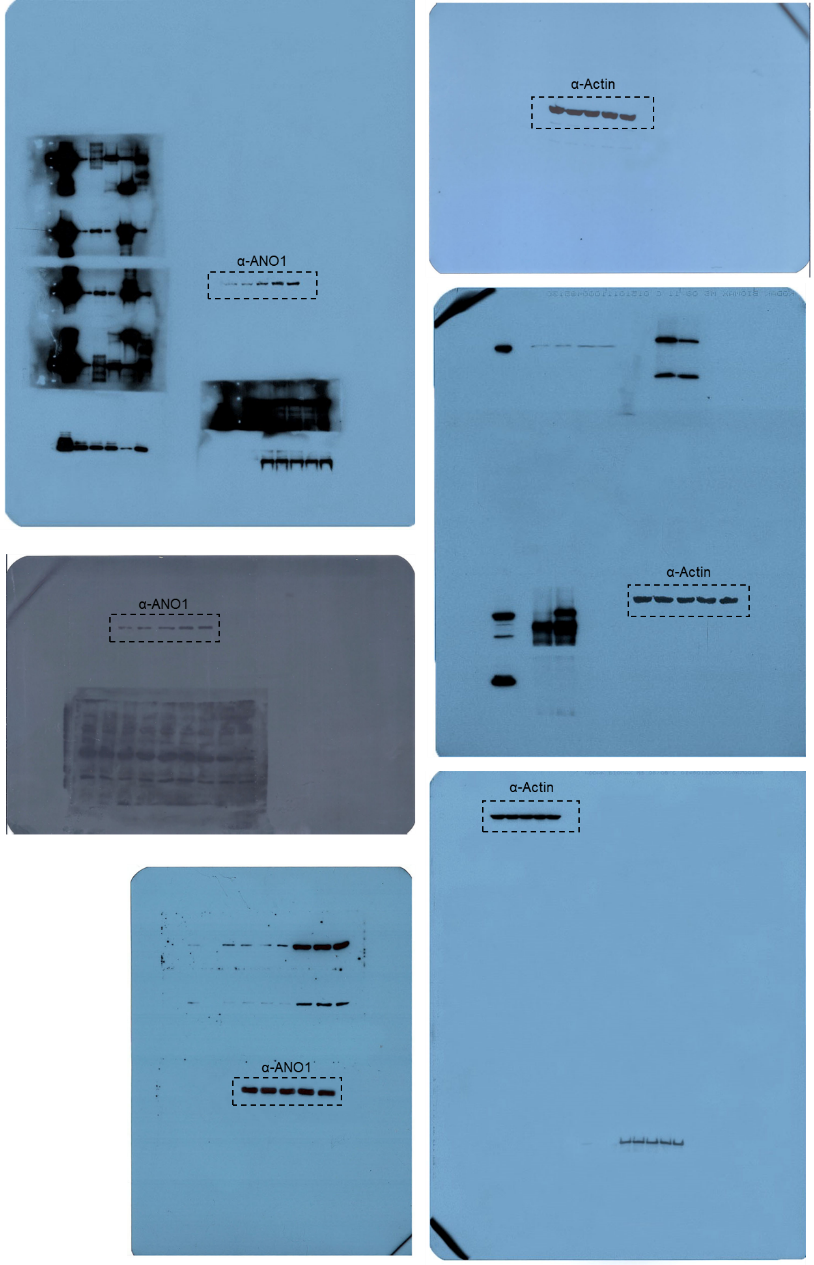
**

**The uncropped original image in Figure 3**

**3D**

**
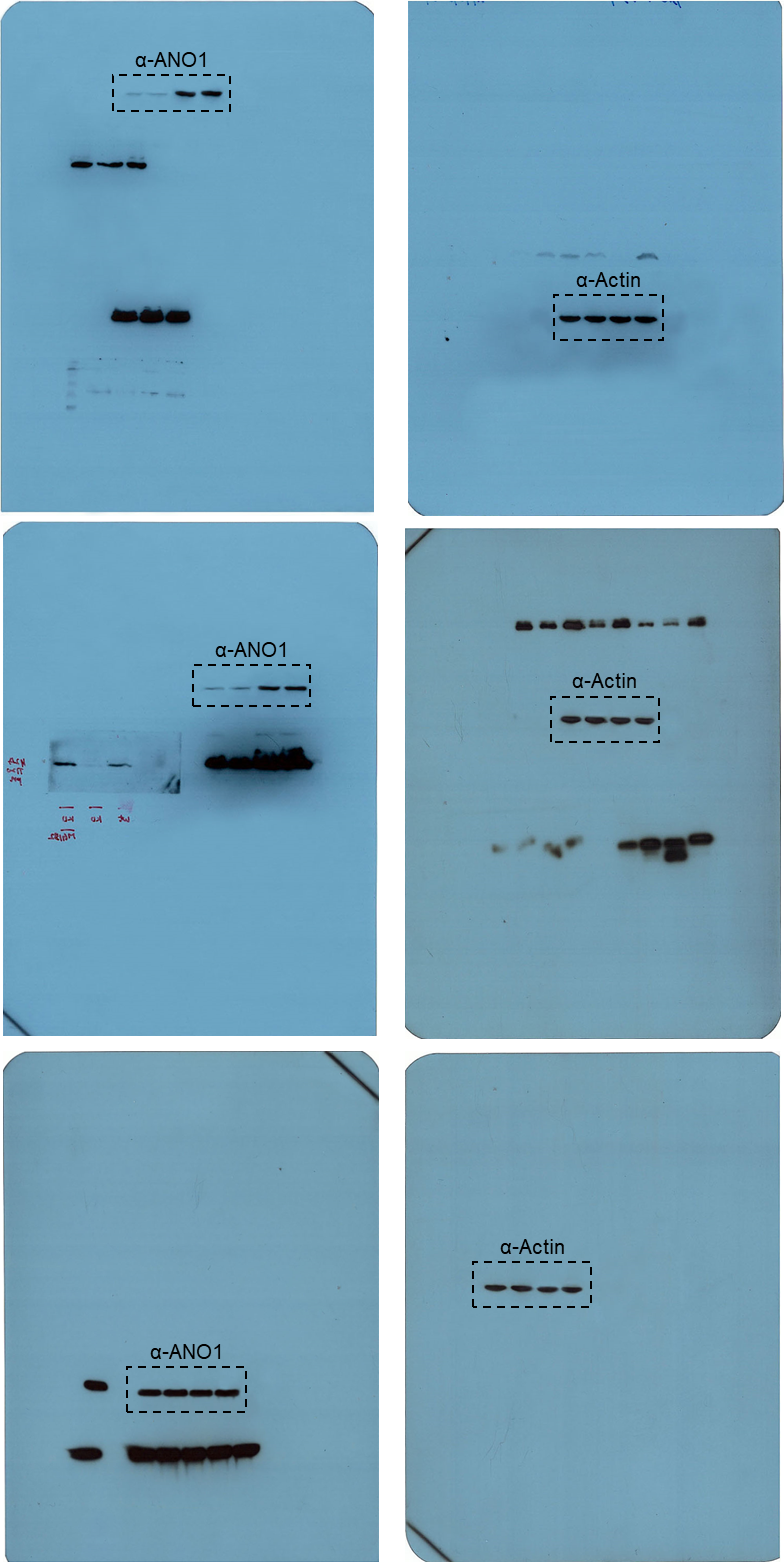
**

**The uncropped original image in Figure S1**

**1B**

**
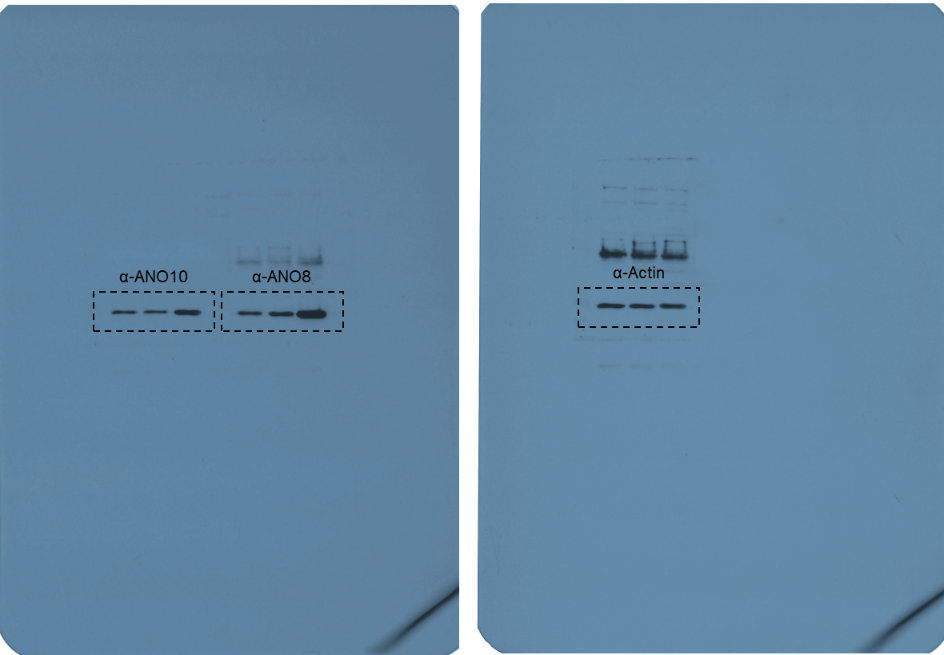
**

**The uncropped original image in Figure S5**


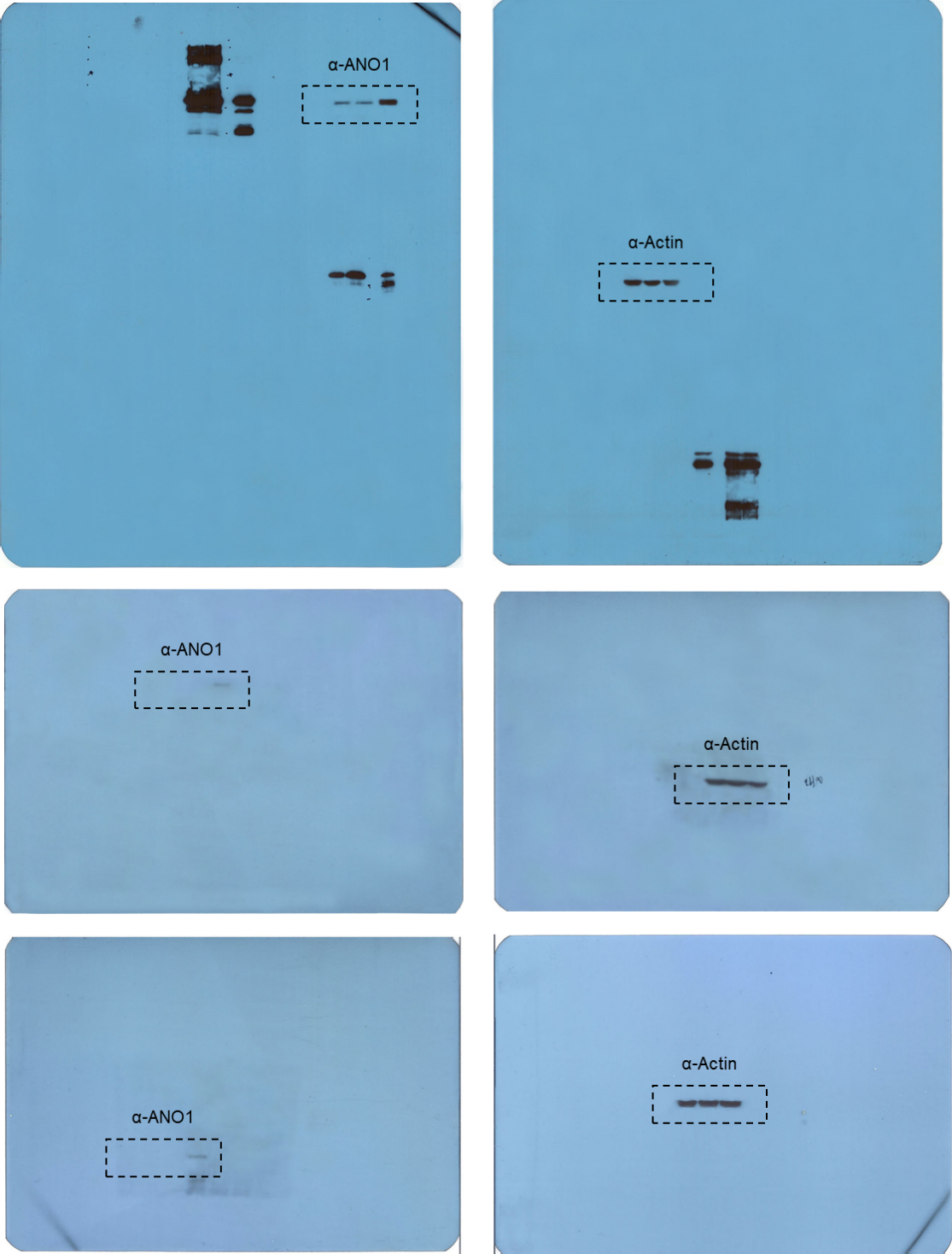

Supplement: Supplementary file 1 — Supplementary Figures. [file 41598_2024_62478_MOESM1_ESM.docx]
