# Supplementary material for: Promoter hypermethylation as a novel regulator of ANO1 expression and function in prostate cancer bone metastasis
Source: Sci Rep. 2024 May 21;14:11595. doi: 10.1038/s41598-024-62478-1 (PMC11109272; doi:10.1038/s41598-024-62478-1)
Supplement: Supplementary file 2 — Supplementary Table 1. [file 41598_2024_62478_MOESM2_ESM.docx]

**Supplementary Table 1.** List of the primers used in RT-qPCR.

| Primers | Forward (5'-3') | Reverse (5'-3') |
| --- | --- | --- |
| ANO1 | AACCACACCCTCTCCTCCTT | CTTTGGTGTTGTGGTGGTTG |
| ANO8 | TTCCCAGACACGACCGATGA | TACGTGGCGGTGACAAAGAAG |
| ANO10 | CGTCTGGCATCGTGATTCAG | GCAAACCGAGTGTACCAGGT |
| CBL | agcagcccattagtaggtcc | gtgtcctcttcaccctcaca |
| CDH1 | acaacaagcccgaattcacc | ggtgttcacatcatcgtccg |
| EZR | agttgatgcccttggactga | tcaggtgccttcttgtcgat |
| EGFR | aggtgaaaacagctgcaagg | ccagaaggttgcacttgtcc |
| VR1 | acagctactacaagggccag | gccgccctttggttttctta |
| CFTR | AAGCTGTCAAGCCGTGTTCT | CTGCCTTCCGAGTCAGTTTC |
| KIT | gctgttatgcactgatccgg | gtgtatttgccggtgttggt |
| PLCG1 | tcaagtgtgcagtcaaagcc | ctccctcttgcttctccaca |
| TRVP4 | ctctcaccgcctactaccag | gaacaggaccccagtgaaga |
| CTSK | ACGGAGGCATTGACTCTGAAGATG | GGAAGCACCAACGAGAGGAGAAAT |
| OSCAR | GCTGGTAACGGATCAGCTCCCCAGA | CCAAGGAGCCAGAACCTTCGAAACT |
| NFATC1 | GAGACAGACATCGGGAGGAAGA | GTGGGATGTGAACTCGGAAGA |
| MMP-9 | CAATCCTTGCAATGTGGATG | AGTAAGGAAGGGGCCCTGTA |
| GAPDH | GAAGGTGAAGGTCGGAGTC | GAAGATGGTGATGGGATTTC |
